# Association of Employee Engagement Factors and Turnover Intention Among the 2015 U.S. Federal Government Workforce

**DOI:** 10.1177/2158244020931847

**Published:** 2020

**Authors:** Ilana O. McCarthy, Ramal Moonesinghe, Hazel D. Dean

**Affiliations:** 1 Centers for Disease Control and Prevention, Atlanta, GA, USA

**Keywords:** health manpower, employment, job satisfaction, career mobility, Federal Employee Viewpoint Survey

## Abstract

Employee turnover is a major challenge facing the federal workforce, which has lost more employees to voluntary turnover than any other form of turnover. This study determined the associations between engagement, demographic factors, and voluntary turnover intention by analyzing 2015 Federal Employee Viewpoint Survey data. The findings indicate that employees with higher engagement levels are less likely to report an intention to leave their jobs than those with lower engagement levels. All engagement factors—perceptions of supervisors, leaders, and intrinsic work experience—are independently associated with turnover intention. Demographics also influenced turnover intention; being younger, male, and in a supervisory role and having a higher education level and shorter tenure were more likely to indicate turnover intention. Increasing employee engagement can have a positive effect on retaining a productive federal workforce. To retain an effective federal workforce, human capital management practices are needed to optimize factors that reduce turnover intention.

Retaining an engaged and competent U.S. federal workforce is essential for federal agencies to fulfill their missions. The federal government lags behind the private sector in employee engagement, 62% versus 77% (Partnership for Public Service, 2019). This is important because engaged employees are more productive and are less likely to leave the workforce, or turnover (Byrne et al., 2017; Jin & Park, 2016; Liss-Levinson et al., 2015; Schaufeli & Bakker, 2004; Trahant, 2009). The federal government is already at risk for higher levels of employee turnover (Cho & Lewis, 2012).

Voluntary turnover, a type of turnover in which an employee chooses to resign from a job (U.S. Bureau of Labor Statistics [BLS], 2017), can have detrimental effects on an organization (Bertelli, 2007; Hur, 2013). It can result in a loss of internal working knowledge, an interruption in work activities and productivity, increased costs associated with finding a suitable replacement, and a disruption to team work cohesion; ultimately, it can have a negative impact on organizational performance (Park & Shaw, 2013; Shaw, 2011; Strober, 1990). Previous meta-analytic reviews confirmed that as voluntary turnover rates increased, employee work attitudes, work productivity, customer satisfaction, and the organization’s financial performance decreased (Heavey et al., 2013; Park & Shaw, 2013). Both human capital (i.e., the knowledge and skills of experienced employees) and social capital (i.e., the social bonds and collective organizational goals) are negatively affected by voluntary turnover (Park & Shaw, 2013; Shaw et al., 2005; Strober, 1990). The type of industry influences the strength of this relationship: “The results show a relatively stronger negative relationship between voluntary turnover rates and organizational performance in industries with higher human capital emphasis (e.g., service industries) compared with industries with lower human capital emphasis (e.g., manufacturing)” (Park & Shaw, 2013, p. 282).

The U.S. Federal Government is a public service industry (BLS, 2019) and invests heavily in human capital. On the basis of previous turnover research, turnover would likely have more detrimental effects on governmental performance than other nonservice organizations. This exemplifies the necessity of identifying the causes of turnover, such as a lack of engagement, and remediate them before organizational performance suffers. To better understand these predictors, we examined organizational climate survey data collected by the 2015 U.S. Federal Employee Viewpoint Survey (FEVS) with the aim of identifying employee factors, including demographics and engagement levels, associated with turnover intention among the federal workforce.

## The Federal Workforce and Turnover

In recent decades, the federal workforce has fluctuated in size from a high of 2.25 million in 1990 to a low of 1.78 million in 2000 (U.S. Office of Personnel Management [OPM], 2015e). From 2011 to 2018, the workforce decreased from 2.14 million to 2.08 million employees (OPM, 2018). This decrease in the federal workforce is predicted to continue until at least 2024. BLS (2015) projections indicate that the federal workforce will decrease by approximately 400,000 employees during 2014–2024. According to OPM’s (2018) federal workforce data, resignations or voluntary turnover accounted for 36% (577,264/1,588,725) of all separations from 2011 to 2018. Among the six categories, resignations were the most frequent cause of separations, followed by retirements at 32% (504,237/1,588,725).

To investigate turnover in the federal workforce, the Partnership for Public Service (2014) examined the characteristics of federal employees who had left federal service. They reported that the highest rates of attrition were among entry-level employees (General Schedule [GS] Grades 1–9) and those in the Senior Executive Service (8% and 11%, respectively) during 2013 (Partnership for Public Service, 2014). In addition, employees with <10 years of service accounted for one third of all separations from 2002 to 2012 (Partnership for Public Service, 2014). Losing the most experienced employees to turnover, the senior executives—the leaders of agencies—and the newer entry-level employees can result in organizational setbacks. First, the agency suffers a loss of knowledge, skills, and strategic direction when an executive leaves (Hambrick & Mason, 1984). Second, executive turnover might cause organizational instability and affect performance by redefining the mission, work processes, and policies, thus disrupting the work environment for employees (Boyne et al., 2011; Park & Shaw, 2013). Third, when entry-level employees and those with shorter tenure turnover, the talent pipeline is reduced. Grooming younger employees for higher-skilled positions is a part of successful knowledge transfer and succession planning (Calo, 2008). High turnover rates pose long-term challenges to organizations and can indicate that a systemic problem exists with employee satisfaction, relationships with supervisors, or the leadership capabilities of top management.

## Turnover Determinants

### Employee characteristics.

Employee characteristics have been associated with turnover intention. Age, sex, and tenure have been consistently associated with turnover intention in that younger employees, male employees, and those with fewer years invested in an organization are more likely to indicate intention to leave their jobs than their older, female, and longer tenured counterparts (Bertelli, 2007; Ertas, 2015; Leider et al., 2016; Liss-Levinson et al., 2015; Moynihan & Landuyt, 2008; Pitts et al., 2011; Pourshaban et al., 2015). These disparities by age and tenure in an organization increase among employees born during 1977–1995 (i.e., referred to as millennials). Ertas (2015) reported that millennials are five times more likely to report turnover intention than their older colleagues. Higher education is also associated with greater intention to change jobs in public health (Moynihan & Landuyt, 2008; Pourshaban et al., 2015).

### Employee attitudes.

Employee attitudes (e.g., satisfaction and engagement) are inversely correlated with turnover intention. Substantial evidence indicates that job and pay satisfaction are strong factors that influence both public- and private-sector employees not to change jobs. Employees who reported greater job satisfaction were less likely to intend to leave their organizations (Ertas, 2015; Heavey et al., 2013; Jin & Park, 2016; Kim & Fernandez, 2017; Leider et al., 2016; Luz et al., 2016; Moynihan & Landuyt, 2008; Pitts et al., 2011; Pourshaban et al., 2015), whereas dissatisfaction with pay increased the likelihood of turnover intention (Bertelli, 2007; Liss-Levinson et al., 2015; Luz et al., 2016; Pourshaban et al., 2015). Furthermore, perceptions of job embeddedness (Mitchell et al., 2001), job empowerment (Kim & Fernandez, 2017; Moynihan & Landuyt, 2008), and job control (Rodwell et al., 2011) influence employee turnover. We consider these perceptions similar to those measured in the Intrinsic Work Experience subfactor within the FEVS Employee Engagement Index (EEI) which “captures employee feelings of motivation and competency relating to their role in their workplace” (OPM, 2016a). Thus, our first hypothesis is as follows:

**Hypothesis 1:** Intrinsic Work Experience will be negatively associated with turnover intention at both the organization and federal government levels.

Research involving both the private- and public-sector workforces reveals that high employee engagement is associated with low turnover intention (Bogaert et al., 2019; Byrne et al., 2017; Jin & Park, 2016; Liss-Levinson et al., 2015; Schaufeli & Bakker, 2004). The majority of the research on engagement and turnover intention in the public sector is derived from state and local government employees, rather than federal government employees. Research examining employee engagement, comprehensively measured by the EEI, among federal government employees by using the FEVS is limited to Byrne et al.’s (2017) work (Fernandez et al., 2015). Although Byrne et al. (2017) reported a negative correlation between federal employee engagement and turnover intention, demographic variables (e.g., age, tenure, and education) were not considered. Our research is the first to investigates the effects of age and other demographic variables and their association with engagement (EEI score and subfactors) and turnover intention among federal employees.

### Organizational and relational factors.

Organizational and relational factors are also indicators of turnover intention. Negative correlations have been reported between turnover and person–organization fit, positive supervisory relations, positive coworker support, organizational commitment, and positive attitudes toward the organization (Heavey et al., 2013; Luz et al., 2016; Moynihan & Pandey, 2007). These organizational and relational factors are also independently related to engagement and satisfaction. An employee’s relationship with his or her supervisor influences overall job satisfaction and, subsequently, turnover intention. Supervisory support for employee development, a positive working relationship, and being treated with respect are determinants of job satisfaction (Pourshaban et al., 2015). Similarly, supportive supervisors and perceived organizational support for learning and growth are positively related to employee engagement and employee satisfaction (Jin & McDonald, 2017; Jin et al., 2016). Using the FEVS, Pitts et al. (2011) developed a two-model structure to study predictors of turnover intention on two levels, leaving an organization and leaving the federal government. While other predictors including satisfaction were significant on both levels, relationship with supervisor was only statistically significant in the model for leaving the organization for another federal position. In line with previous research, we expect the second subfactor of the EEI, Supervisors, which “describes the interpersonal relationship between employee and supervisor, including trust, respect, and support” to influence turnover intention (OPM, 2016a). Our second hypothesis is as follows:

**Hypothesis 2:** Supervisors will be negatively associated with turnover intention at the organization level.

This extends to leadership as well—having leaders who exemplify team-oriented behaviors, inspire and motivate employees, and demonstrate integrity results in increased employee engagement and satisfaction (Trottier et al., 2008; Xu & Cooper Thomas, 2011). Leaders have the ability to drive performance, create organizational culture changes, and ultimately empower employees to achieve the organization’s mission. Certain leadership styles, such as transformational leadership, have been associated with employees indicating increased work engagement, receiving higher performance ratings, and executing enhanced job performance (Breevaart et al., 2016; Ghafoor et al., 2011; Vincent-Höper et al., 2012). The third subfactor of the EEI, Leaders Lead “reflects employee perceptions of the integrity of leadership, as well as leadership behaviors such as communication and workforce motivation” (OPM, 2016a), which are elements of transformational leadership. Thus, our third hypothesis is as follows:

**Hypothesis 3:** Leaders Lead will be negatively associated with turnover intention at both the organization and federal government levels.

## The Present Study

In the existing literature, substantial evidence demonstrates that more satisfied employees are less likely than less satisfied employees to report an intention to leave government employment (Ertas, 2015; Leider et al., 2016; Pitts et al., 2011; Pourshaban et al., 2015). Furthermore, employee engagement is a prerequisite for job satisfaction, and thus, interventions that improve engagement can lead to improvements in job satisfaction (Jin & Park, 2016; OPM, 2015a; Saks, 2006) and result in less turnover. However, we do not know the strength of association between employee engagement and turnover intention when the effects of age and tenure are considered.

In this study, we explore the association between employee engagement and turnover intention, adjusted for demographic variables. Our study adds to the existing literature in two ways: (a) We extend Pitts et al.’s (2011) research by investigating employee engagement by using his two-model structure, and (b) we extend Byrne et al.’s (2017) analysis by examining demographic variables and their impact on turnover intention. Literature on demographic and attitudinal factors in the federal workforce leading to turnover is lacking; this analysis can reveal groups in the federal government that are vulnerable to turnover intention and where human capital management efforts are most needed for engaging and retaining the U.S. federal workforce.

## Data and Methods

We used data from the 2015 FEVS to investigate the association among age, tenure, and employee engagement, and turnover intention among federal government employees. OPM (2015c) collects FEVS data to measure “employees’ perceptions of conditions within their agencies which contribute to their organization’s success” (p. v). For the 2015 FEVS, OPM (2015d) used a stratified sampling technique in which 903,060 federal employees of the 1,837,060 eligible employees were included in the final sample, representing 82 agencies in the federal government. The sampled employees were invited to participate in a secure online survey throughout a 6-week data collection period (OPM, 2015d).

Our analysis was conducted by using the 2015 FEVS public-release data file (OPM, 2015b) provided by OPM; no changes were made to alter the file for analytic purposes. We chose 2015 FEVS data because it included key demographic variables that were excluded in later years from the public-release data files. All agencies and subagencies were used regarding selected demographic variables, including age, tenure, sex, education level, supervisory status, and employee engagement level (i.e., EEI score) and the effect of the variables on turnover intention. These analyses were conducted at the individual level.

### Turnover Intention

Although we cannot estimate actual turnover with the FEVS data, turnover intention is a common proxy indicator for actual turnover in the public sector (Bertelli, 2007; Cho & Lewis, 2012; Ertas, 2015; Pitts et al., 2011). To categorize turnover intention, FEVS asks, “Are you considering leaving your organization within the next year and if so, why?” The answer options provided in the public use data set were as follows: (A) *No*; (B) *Yes, to take another job within the federal government*; (C) *Yes, to take another job outside the federal government*; or (D) *Yes, other*, which included responses related to retirement. OPM merged the *Yes, other* and *Yes, to retire* answer options to protect the identity of survey respondents. For the purpose of this study, this merged category was excluded from the analysis. On the basis of previous studies (Ertas, 2015; Pitts et al., 2011), turnover intention was created as a two-level variable in two analytic steps. First, turnover intention was coded dichotomously with *Yes* or *No* for intention to leave. Second, leaving the federal government was coded separately as taking another job either within or outside the federal government. To be included in the second-level coding, the respondent must have reported *Yes* for intention to leave.

### Statistical Analysis

SAS version 9.3 (SAS Institute, Inc., Cary, North Carolina) was used for statistical analyses. For the 2014 FEVS, OPM (2014) conducted a factor analysis that resulted in three factors of 15 items for the EEI score.

The first factor, Leaders Lead (perceptions of the integrity of leadership, as well as leadership behaviors, e.g., communication and workforce motivation), was based on the following questions:

Q53: In my organization, senior leaders generate high levels of motivation and commitment in the workforce.
Q54: My organization’s senior leaders maintain high standards of honesty and integrity.
Q56: Managers communicate the goals and priorities of the organization.
Q60: Overall, how good a job do you feel is being done by the manager directly above your immediate supervisor?
Q61: I have a high level of respect for my organization’s senior leaders.

The second factor, Supervisors (reflects the interpersonal relationship between worker and supervisor, including trust, respect, and support), was based on the following questions:

Q47: Supervisors in my work unit support employee development.
Q48: My supervisor listens to what I have to say.
Q49: My supervisor treats me with respect.
Q51: I have trust and confidence in my supervisor.
Q52: Overall, how good a job do you feel is being done by your immediate supervisor?

The third factor, Intrinsic Work Experience (reflects the employees’ feelings of motivation and competency relating to their role in the workplace), was based on the following questions:

Q3: I feel encouraged to come up with new and better ways of doing things.
Q4: My work gives me a feeling of personal accomplishment.
Q6: I know what is expected of me on the job.
Q11: My talents are used well in the workplace.
Q12: I know how my work relates to the agency’s goals and priorities.

We conducted an exploratory factor analysis using the identical 15 items, for the 2015 FEVS data. We used the varimax (orthogonal) rotation method to compute factor loadings. The meanings of the rotated factors were inferred from the measures statistically significantly loaded on their factors. Factor loadings >0.6 in absolute value were considered significant. Next, factor scores were calculated for each respondent on the basis of these factor loadings.

Next, a multivariate logistic regression model was fit to examine the association between intention to leave one’s organization within the coming year, excluding those indicating other or retirement, and the calculated factor scores. The analysis was extended to examine the association between the intention to leave one’s organization for a job within the federal government, of those who indicated intention to leave, with factor scores using the same regression model.

### Independent Variables

We used factor scores and employee demographics as independent variables in the regression model. Employee demographics included age group, federal tenure, highest level of education completed, sex, and supervisory status. The age group variable was coded into four categories: <40, 40–49, 50–59, and ≥60 years. Federal tenure was categorized by length of time in the federal government, excluding military service. Three categories were provided in the data set: ≤5, 6–14, and ≥15 years of federal service. Education level was coded into three categories: less than a bachelor’s degree, a bachelor’s degree, and a post-bachelor’s degree. Finally, supervisory status and sex were dichotomously coded.

## Results

**Figure 1.**
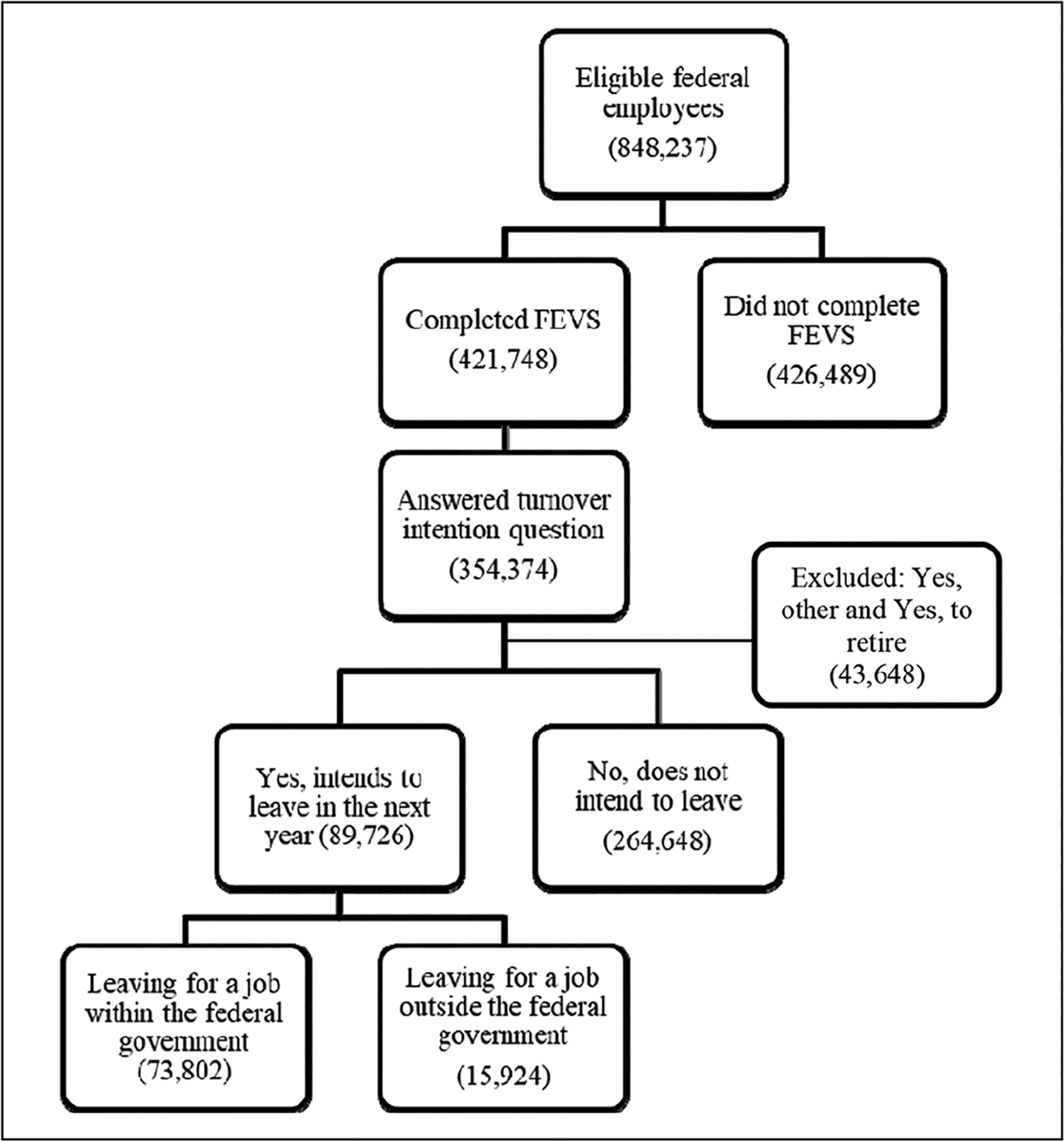
Federal Government employee completion of 2015 Federal Employee Viewpoint Survey (FEVS) questions related to turnover intention.

During 2015, a total of 848,237 employees among the final sample received the FEVS; 54,823 were determined ineligible at the close the survey (OPM, 2015d). Of the 848,237 eligible employees, 421,748 U.S. federal employees completed the FEVS (Figure 1) for a 50% response rate (OPM, 2015b). Table 1 displays the turnover intentions of the sample and the demographic characteristics by turnover intention status of either leaving the organization or leaving federal government. In the 2015 FEVS, 25% (89,726/354,374) of employees in our sample indicated that they plan to leave their organization within the year after the survey. Of the quarter of employees who intended to leave their organization, 18% (15,924/89,726) reported that they would leave for a job outside the federal government, and 82% (73,802/89,726) plan to leave for another federal position. Differences existed between demographic variables and turnover intention. For example, 33% (26,382/78,837) of employees in the youngest age category (<40 years) reported a turnover intention of leaving their organization, and another 22% (5,736/26,382) reported they planned to leave the federal government. Intention to leave the organization decreased with increasing age. However, the oldest age group (ages ≥60 years) had the highest rate (25%; 1,383/5,623) of intention to leave for a job outside of the federal government, compared with the other age groups. Intention to leave the organization or to leave the federal government increased with increasing education and decreased with increasing tenure. Descriptive statistics for all variables are reported in Table 1.

The exploratory factor analysis of the 15 items revealed that three eigenvalues of the correlation matrix were >1.0, indicating that three unobserved attitudes and opinions about employee engagement (factors) accounted for the majority of the variability in the 15 measures between employees. Table 2 lists rotated (varimax) factor loadings for the 15 measures. These three factors (supervisor, leaders lead, and intrinsic work experiences) explained 76% of the variation in the 15 measures when using a cutoff point of 0.6, and were identical to the three factors obtained by OPM for their factor analysis of the 2014 FEVS data.

Next, factor scores were calculated for each of the three factors for employees. The factor scores ranged from −5.5 to 3.7, and these factor scores were treated as independent continuous variables in the multivariate logistic regression to determine how these three factors influence intention to leave one’s organization, excluding those who intended to retire or selected other. Table 3 lists the estimates of odds ratios (ORs) and 95% confidence intervals (CIs) for all the independent variables considered. All the ORs were significant at the .05 level. Intentions to leave one’s organization were 39%, 45%, and 46% less likely for each unit increase in scores of Factor 1 (Supervisors), Factor 2 (Leaders Lead), and Factor 3 (Intrinsic Work Experience), respectively. Thus, our three main hypotheses are supported at the organization level; engagement factors are negatively associated with turnover intention. Odds of intention to leave one’s organization declined with increasing age. For example, odds of intention to leave one’s organization for employees in age groups 40–49, 50–59, and ≥60 years were 94%, 75%, and 36% times, respectively, the odds of intention to leave for the age group <40 years. Similarly, the odds of intention to leave one’s organization declined with increasing length of time in the federal government. Employees with federal tenures of 6–14 and ≥15 years were 17% and 50%, respectively, less likely to leave their organizations, compared with employees with federal tenure of ≤5 years. In contrast, employees with a higher level of education were more likely to leave their organizations. Employees with a bachelor’s degree and employees with a post-bachelor’s degree were 16% and 13%, respectively, more likely to intend to leave their organizations, compared with employees with less than a bachelor’s degree. Female employees were 6% less likely to intend to leave their organizations, compared with males, whereas the odds of supervisors leaving their organizations were 9% higher, compared with nonsupervisors.

The next logistic regression determined how the three calculated factors influence the intention to leave one’s organization for a job within the federal government. In this analysis, only those who reported an intention to leave were included and then grouped by intention to leave for a job within the federal government or intention to leave for a job outside of the federal government. Table 4 provides the ORs and 95% CIs for all the independent variables considered. Variable for scores of Factor 1 (Supervisors) was nonsignificant at the .05 level. Odds for intention to leave one’s organization for a job within the federal government increased with increasing scores of Factor 2 (Leaders Lead) and Factor 3 (Intrinsic Work Experience). This finding supports our two hypotheses at the federal government level, whereby Intrinsic Work Experience and Leaders Lead are negatively associated with turnover intention. Intention to leave for a job within the federal government were 11% and 44% more likely for each unit increase in scores of Factor 2 (Leaders Lead) and Factor 3 (Intrinsic Work Experience), respectively. Odds of leaving for a job within the federal government for the age groups 40–49 and 50–59 years were 44% and 57%, respectively, higher, compared with the age group <40 years. However, employees aged ≥60 years were 11% less likely to leave for a job within the federal government. Employees with federal tenure of 6 to 14 and ≥15 years were 16% and 22%, respectively, more likely to leave for a job within the federal government, compared with employees with federal tenure of ≤5 years. Employees with a bachelor’s degree and employees with a post-bachelor’s degree were 18% and 49%, respectively, less likely to leave for a job within the federal government, compared with employees with less than a bachelor’s degree. Female employees were 54% more likely to leave for a job within the federal government, compared with males, and supervisors were 27% less likely to leave for a job within the federal government, compared with nonsupervisors.

## Discussion

This study determined the strength of the association between factors that account for the variability of employee engagement and turnover intention at two levels (i.e., employee intention to leave federal employment or intention to change federal jobs). First, the study confirms with 2015 data the results of OPM’s factor analysis of the subindices within the EEI score; the factors were identical to the three factors (Leaders Lead, Intrinsic Work Experience, and Supervisors) obtained by OPM for the 2014 FEVS (OPM, 2014). Second, in the first logistic regression model, the analysis indicates that the association between employee engagement and intention to leave the organization was statistically significant. We found support for all three of our hypotheses; with increasing ratings of each factor, Supervisors, Leaders Lead, and Intrinsic Work Experience, employees were less likely to indicate turnover intention. That is, employees who perceived their supervisors as supportive, their leaders as honest, and their work as motivating were less likely to want to leave their organizations. Intrinsic Work Experience had the greatest influence on turnover intention, whereas Supervisors had the least.

Overall, the findings indicate that a quarter of all federal employees indicate a form of turnover intention—intention to leave one’s organization during the year after the survey. Of that group, approximately one fifth indicated an intention to leave the federal government. However, employees aged <40 or ≥60 years disproportionately reported an intention to leave the federal government for an external position. The first of those two findings was expected; that is, previous research reported that younger employees were more likely to leave the public sector (Ertas, 2015; Leider et al., 2016; Moynihan & Landuyt, 2008; Pitts et al., 2011; Pourshaban et al., 2015). Younger employees have fewer years vested in the government and might find private-sector employment, including pay, more attractive. Federal managers can implement evidence-based strategies to retain younger employees by providing merit-based rewards and fair performance appraisals (Cho & Lewis, 2012).

The second finding is new—increased age has been consistently inversely related to turnover intention in government service (Ertas, 2015; Leider et al., 2016; Moynihan & Landuyt, 2008; Pitts et al., 2011; Pourshaban et al., 2015). In this sample of 2015 federal employees, that is not the case. Additional research is needed to determine whether this is a growing trend among older employees in the federal government.

In both models, increasing tenure reduced the odds of turnover intention. This result corroborates previous research (Leider et al., 2016; Liss-Levinson et al., 2015; Moynihan & Landuyt, 2008; Pitts et al., 2011). As tenure increases in the federal government, employees are more invested in their positions; their benefits have improved; and retirement and access to federal pensions become more salient. The U.S. civil service retirement system has an impact on retaining employees in the federal government, because the pension incentive increases with increasing years of service. This is most noticeable with midcareer employees, those with 11 to 29 years invested in federal service; specifically, midcareer private-sector employees have a separation rate that is eight times higher than midcareer federal employees (Falk & Karamcheva, 2017).

Two additional results are noteworthy: supervisors and those with higher educations had greater odds of leaving the organization and leaving the federal government. In contrast with previous research (Moynihan & Landuyt, 2008; Pourshaban et al., 2015), we determined that supervisors were more likely to indicate turnover intention. This is perhaps a result of the capped GS pay system in the federal government; in 2018, employees at the highest grade and step (GS-15, Step 10) maxed out their pay at $136,659 base pay. Private-sector employment might be financially more attractive to supervisors in the federal government.

Research also indicates that private-sector employment is more financially beneficial to those with higher education levels (Falk, 2017). In line with previous research, we determined that education level is a predictor of turnover intention in the public sector (Liss-Levinson et al., 2015; Moynihan & Landuyt, 2008). This might be attributable to the wage differences by education level in the federal government versus the private sector. A Congressional Budget Office study reported that federal employees with a master’s degree earned 7% less in wages, compared with their private-sector counterparts (Falk, 2017). This wage gap dramatically increased for federal employees with a professional or doctorate degree; they earned 24% less than those in the private sector (Falk, 2017). Thus, highly educated federal employees might choose to leave the federal government for the financial benefit of private-sector pay. This result was consistent in both logistic regressions; that is, higher education predicted turnover intention within 1 year and intent to find a job outside the federal government. Retaining supervisors and those with advanced degrees should be a priority for federal managers. Considering alternative benefits such as flexible work schedules, teleworking, and the use of cash awards can be tools to retain these employees.

In the second logistic regression model, investigating intention to leave for another federal government position, the Supervisor factor of the EEI score was no longer statistically significant. Although Pitts et al. (2011) used a different indicator for relationship with supervisors, this result was the same—statistically significantly related to turnover intention in the first logistic regression and insignificant in the second logistic regression. We hypothesized replicating this finding; nonetheless, it is interesting and future research is needed to determine how supervisors influence employee turnover. In our study, perceptions of work experience and leaders are the most influential factors on both levels of turnover intention. In the leaving federal government model, women were 54% more likely to indicate intention to stay within the federal government by acquiring an internal job; similar research reveals women are less likely than men to leave their public-sector positions (Bertelli, 2007; Moynihan & Landuyt, 2008).

The federal government can implement strategies to positively influence employee engagement and to reduce the likelihood of federal employees separating from their postings or leaving the federal government for the private sector. One strategy for addressing deficits in employee engagement is to intervene at the level of engagement drivers (U.S. Government Accountability Office [GAO], 2015; OPM, 2016b). Examples of such drivers include providing constructive performance feedback, promoting training and development opportunities, and supporting collaborations and communication among team members (GAO, 2015; OPM, 2016b). The key to increasing employee engagement is to demonstrate value for employees’ work, their opinions related to their work, and their career growth (GAO, 2015). Furthermore, GAO (2015) reported that performance conversations were the best predictor of employee engagement. Federal administrators can create an organizational environment that fosters employee engagement and reduce turnover by providing supervisory trainings on performance conversations and employee development.

Another strategy is to target the actual subindices or specific items within the EEI. To create an organizational culture that promotes the most influential engagement factors, agencies can encourage employees to have autonomy over their work and to connect their accomplishments to the agency’s priorities—this will likely lead to increases in the Intrinsic Work Experience subindex and reductions in turnover intention. Rodwell et al. (2011) and Moynihan and Landuyt (2008) similarly report that job control or job autonomy is a substantial predictor of intentions to quit, whereas increasing job control resulted in fewer quit intentions. Federal managers can receive training on empowering employees to increase their job control and autonomy; these empowering behaviors can result in employee psychological empowerment, work engagement, and a reduced turnover intention (De Klerk & Stander, 2014). Furthermore, to improve Leaders Lead competencies, agency and subagency leaders can be provided with opportunities to hone their leadership skills (e.g., exhibiting integrity, communicating effectively, and motivating employees to work toward fulfilling the agency’s mission). Xu and Cooper Thomas (2011) determined that employee engagement correlated with specific leadership behaviors—supporting the team, performing effectively, and demonstrating integrity. These leadership and organizational changes can cultivate engagement and increase long-term retention.

Evidence exists that improvements in work conditions and perceptions can change turnover intention over time. A recent study measured intent to leave in 2014 and 2017 among public health workers (Bogaert et al., 2019). Bogaert et al. (2019) reported that workers who had intended to leave in 2014 but who were still in their positions in 2017 indicated improvements in engagement and satisfaction and no turnover intention in 2017. Workplace interventions to improve satisfaction and engagement (e.g., enhancing supervisory relationships, ensuring fair and equitable pay, and increasing job embeddedness) can result in employee retention.

Our study had certain limitations. First, the data were derived from the 2015 FEVS, which is voluntary. Thus, non-response bias might exist in the data set used if more engaged employees were more or less likely than less engaged employees to complete the survey. Second, only respondents who answered all 20 questions—the 15 that are included in the EEI score and the turnover intention and demographic variables—were included in the logistic regressions; and missing item responses might have been nonrandom. Third, a recent article stated that turnover intention might not accurately describe actual turnover rate at the organizational level (Cohen et al., 2016). Our study investigated the strength of independent association between employee engagement or demographics and turnover intention at the individual level. As a cross-sectional study, it cannot predict actual organizational turnover; it can only measure the strength of association of those variables with individual turnover intention at the time of the survey. Further research is needed to understand the association between turnover intention and actual turnover rate at the individual level. Finally, although these analyses indicate a strong association between engagement factors and the self-reported intention to leave a federal organization and the federal government, other factors likely affect employee engagement and turnover intention that are not included in our study.

## Conclusion

Our research provides new insights about the associations among turnover intention, demographic factors, and engagement factors, including employees’ perceptions of leaders, supervisors, and their work experiences. This is the first study to use calculated factor scores from the EEI to predict turnover intention, while adjusting for demographic variables. Specifically, the results indicate employees with higher education levels and in supervisory positions were more likely to indicate intention to leave the federal government. These findings demonstrate the utility of using FEVS data to examine turnover intention in the federal government. Similar methods can be used to conduct studies within federal organizations to understand turnover intention and to inform and tailor organizational response. Furthermore, federal managers can utilize additional organizational climate and engagement surveys, and exit surveys to determine specific reasons why employees turnover. Targeted efforts that enhance leadership behaviors, encourage employee empowerment in their work, and foster performance conversations are initial steps in engaging the federal workforce. Federal government human resource management practices (e.g., transition planning and such employee benefits as reducing the wage gap) can supplement these efforts to attract and retain an engaged and productive federal workforce.

## Figures and Tables

**Table 1.** Number and Percentage of Respondents to the 2015 Federal Employee Viewpoint Survey Who Reported Turnover Intention Within 1 Year of the Survey, by Demographic Variables.

Dependent variables	No. (%)	
Turnover: leaving the organization	89,726 (25)	
Turnover: leaving the organization for another job within the federal government	73,802 (82)	
Independent variables by turnover intention status	Leaving the organization	Leaving the organization for another job within the federal government
Age group (years)		
<40	26,382 (33)	20,646 (78)
40–49	27,970 (29)	23,573 (84)
50–59	28,590 (22)	24,411 (85)
≥60	5,623 (13)	4,240 (75)
Highest education level attained		
Less than a bachelor’s degree	22,660 (22)	20,002 (88)
Bachelor’s degree	30,135 (25)	25,066 (83)
Post-bachelor’s degree	35,636 (29)	27,648 (78)
Federal employment tenure (years)		
≤5	21,676 (31)	17,115 (79)
6–14	39,693 (30)	32,632 (82)
≥15	27,596 (19)	23,405 (85)
Sex		
Men	47,071 (26)	37,249 (79)
Women	39,748 (24)	34,183 (86)
Supervisory status		
Nonsupervisor	72,562 (27)	60,201 (83)
Supervisor	15,504 (21)	12,242 (79)

**Table 2.** Rotated Factor Loadings for Each of the Three Factors that Accounted for the Majority of the Variability in Responses to Each of the 15 Items in the 2015 Employee Engagement Index.

	Rotated factor loadings		
Employee engagement index item	Factor 1 supervisors	Factor 2 leaders lead	Factor 3 intrinsic work experience
Leaders Lead subindex			
Q53: In my organization, senior leaders generate high levels of motivation and commitment in the workforce.	0.19	**0.84**	0.31
Q54: My organization’s senior leaders maintain high standards of honesty and integrity.	0.23	**0.84**	0.26
Q56: Managers communicate the goals and priorities of the organization.	0.26	**0.68**	0.36
Q60: Overall, how good a job do you feel is being done by the manager directly above your immediate supervisor?	0.34	**0.74**	0.23
Q61: I have a high level of respect for my organization’s senior leaders.	0.20	**0.85**	0.27
Intrinsic Work Experience subindex			
Q3: I feel encouraged to come up with new and better ways of doing things.	0.37	0.40	**0.61**
Q4: My work gives me a feeling of personal accomplishment.	0.22	0.25	**0.77**
Q6: I know what is expected of me on the job.	0.30	0.22	**0.72**
Q11: My talents are used well in the workplace.	0.32	0.34	**0.69**
Q12: I know how my work relates to the agency’s goals and priorities.	0.12	0.26	**0.75**
Supervisors subindex			
Q47: Supervisors in my work unit support employee development.	**0.70**	0.35	0.33
Q48: My supervisor listens to what I have to say.	**0.87**	0.20	0.25
Q49: My supervisor treats me with respect.	**0.87**	0.18	0.23
Q51: I have trust and confidence in my supervisor.	**0.88**	0.26	0.23
Q52: Overall, how good a job do you feel is being done by your immediate supervisor?	**0.86**	0.25	0.20

*Note*. Bold type indicates statistically significant factor loadings >0.6 in absolute value.

**Table 3.** Odds Ratios (ORs) for the Association of Each Engagement Factor or Demographic Variable with Intent to Leave the Organization Within 1 Year after the 2015 Federal Employee Viewpoint Survey.

Engagement factor	OR [95% CI]
Factor 1: Supervisors	0.61 [0.60, 0.61]
Factor 2: Leaders lead	0.55 [0.55, 0.56]
Factor 3: Intrinsic work experience	0.54 [0.54, 0.55]
Effect of independent variables	
Age group (years)	
<40	Ref.
40–49	0.94 [0.92, 0.97]
50–59	0.75 [0.73, 0.77]
≥60	0.36 [0.34, 0.37]
Highest education level attained	
Less than a bachelor’s degree	Ref.
Bachelor’s degree	1.16 [1.13, 1.19]
Post-bachelor’s degree	1.52 [1.48, 1.55]
Federal tenure (yrs)	
≤5	Ref.
6–14	0.83 [0.81, 0.85]
≥15	0.50 [0.49, 0.52]
Sex	
Men	Ref.
Women	0.94 [0.93, 0.96]
Supervisory status	
Nonsupervisor	Ref.
Supervisor	1.09 [1.07, 1.12]

*Note*. Respondents who selected option (D) “Yes, other” (which included responses related to retirement), to the turnover intention categorization question, “Are you considering leaving your organization within the next year and if so, why?,” were excluded from this analysis. CI = confidence interval; OR = odds ratio.

**Table 4.** Odds Ratios (ORs) for the Association of Each Engagement Factor or Demographic Variable With Intent to Leave the Organization for Another U.S. Federal Government Job Within 1 Year of the 2015 Federal Employee Viewpoint Survey, Given That the Respondent Indicated Intent to Leave the Organization Within 1 Year.

Engagement factors	OR [95% CI]
Factor 1: Supervisors	1.00 [0.98, 1.01]
Factor 2: Leaders lead	1.11 [1.09, 1.13]
Factor 3: Intrinsic work experience	1.16 [1.14, 1.18]
Effect of independent variables	
Age group (years)	
<40	Referent
40–49	1.44 [1.37, 1.52]
50–59	1.57 [1.48, 1.66]
≥60	0.89 [0.82, 0.97]
Highest education	
Less than a bachelor’s degree	Ref.
Bachelor’s degree	0.72 [0.68, 0.77]
Post-bachelor’s degree	0.51 [0.48, 0.54]
Federal employment tenure (yrs)	
≤5	Ref.
6–14	1.16 [1.10, 1.22]
≥15	1.22 [1.15, 1.30]
Sex	
Men	Ref.
Women	1.54 [1.48, 1.61]
Supervisory status	
Nonsupervisor	Ref.
Supervisor	0.73 [0.69, 0.76]

*Note*. Respondents who selected option (D) “Yes, other” (which included responses related to retirement), to the turnover intention categorization question, “Are you considering leaving your organization within the next year and if so, why?,” were excluded from this analysis. CI = confidence interval; OR = odds ratio.

## References

[R1] Bertelli AM (2007). Determinants of bureaucratic turnover intention: Evidence from the department of the treasury. Journal of Public Administration Research and Theory, 17(2), 235–258. 10.1093/jopart/mul003

[R2] Bogaert K, Leider JP, Castrucci BC, Sellers K, & Whang C (2019). Considering leaving, but deciding to stay: A longitudinal analysis of intent to leave in public health. Journal of Public Health Management and Practice, 25, S78–S86. 30720620 10.1097/PHH.0000000000000928 PMC6586295

[R3] Boyne GA, James O, John P, & Petrovsky N (2011). Top management turnover and organizational performance: A test of a contingency model. Public Administration Review, 71, 572–581. 10.1111/j.1540-6210.2011.02389.x

[R4] Breevaart K, Bakker AB, Demerouti E, & Derks D (2016). Who takes the lead? A multi-source diary study on leadership, work engagement, and job performance. Journal of Organizational Behavior, 37, 309–325. 10.1002/job.2041

[R5] Byrne ZS, Hayes TL, & Holcombe KJ (2017). Employee engagement using the Federal Employee Viewpoint Survey. Public Personnel Management, 46(4), 368–390. 10.1177/0091026017717242

[R6] Calo TJ (2008). Talent management in the era of the aging workforce: The critical role of knowledge transfer. Public Personnel Management, 37(4), 403–416. 10.1177/009102600803700403

[R7] Cho YJ, & Lewis GB (2012). Turnover intention and turnover behavior: Implications for retaining federal employees. Review of Public Personnel Administration, 32(1), 4–23. 10.1177/0734371×11408701

[R8] Cohen G, Blake RS, & Goodman D (2016). Does turnover intention matter? Evaluating the usefulness of turnover intention rate as a predictor of actual turnover rate. Review of Public Personnel Administration, 36(3), 240–263. 10.1177/0734371×15581850

[R9] De Klerk S, & Stander MW (2014). Leadership empowerment behaviour, work engagement and turnover intention: The role of psychological empowerment. Journal of Positive Management, 5(3), 28–45. 10.12775/jpm.2014.018

[R10] Ertas N (2015). Turnover intentions and work motivations of millennial employees in federal service. Public Personnel Management, 44(3), 401–423. 10.1177/0091026015588193

[R11] Falk JR (2017). Comparing the compensation of federal and private-sector employees, 2011 to 2015. Congress of the United States, Congressional Budget Office. https://www.cbo.gov/publication/52637

[R12] Falk JR, & Karamcheva N (2017). Options for changing the retirement system for federal civilian workers. Congress of the United States, Congressional Budget Office. https://www.cbo.gov/publication/53003

[R13] Fernandez S, Resh WG, Moldogazlev T, & Oberfield ZW (2015). Assessing the past and promise of the Federal Employee Viewpoint Survey for public management research: A research synthesis. Public Administration Review, 75, 382–394. 10.1111/puar.12368

[R14] Ghafoor A, Qureshi TM, Khan MA, & Hijazi ST (2011). Transformational leadership, employee engagement and performance: Mediating effect of psychological ownership. African Journal of Business Management, 5(17), 7391–7401. 10.5897/AJBM11.126

[R15] Hambrick DC, & Mason PA (1984). Upper echelons: The organization as a reflection of its top managers. The Academy of Management Review, 9, 193–206.

[R16] Heavey AL, Holwerda JA, & Hausknecht JP (2013). Causes and consequences of collective turnover: A meta-analytic review. Journal of Applied Psychology, 98(3), 412–453. 23668597 10.1037/a0032380

[R17] Hur Y (2013). Turnover, voluntary turnover, and organizational performance: Evidence from municipal police departments. Public Administration Quarterly, 37(1), 3–35. 10.1108/PIJPSM-01-2014-0006

[R18] Jin MH, & McDonald B (2017). Understanding employee engagement in the public sector: The role of immediate supervisor, perceived organizational support, and learning opportunities. American Review of Public Administration, 47(8), 881–897. 10.1177/0275074016643817

[R19] Jin MH, McDonald B, & Park J (2016). Followership and job satisfaction in the public sector: The moderating role of perceived supervisor support and performance-oriented culture. International Journal of Public Sector Management, 29(3), 218–237. 10.1108/IJPSM-05-2015-0101

[R20] Jin MH, & Park J (2016). Sexual minority and employee engagement: Implications for job satisfaction. Journal of Public and Nonprofit Affairs, 2(1), 3–14. 10.20899/jpna.2.1.3-14

[R21] Kim SY, & Fernandez S (2017). Employee empowerment and turnover intention in the U.S. federal bureaucracy. American Review of Public Administration, 47(1), 4–22. 10.1177/0275074015583712

[R22] Leider JP, Harper E, Shon JW, Sellers K, & Castrucci BC (2016). Job satisfaction and expected turnover among federal, state, and local public health practitioners. American Journal of Public Health, 106(10), 1782–1788. 10.2105/ajph.2016.303305 27552269 PMC5024370

[R23] Liss-Levinson R, Bharthapudi K, Leider JP, & Sellers K (2015). Loving and leaving public health: Predictors of intentions to quit among state health agency workers. Journal of Public Health Management and Practice, 21, S91–S101. 10.1097/PHH.0000000000000317 26422500 PMC4590520

[R24] Luz CMDR, de Paula SL, & de Oliveira LMB (2016). Organizational commitment, job satisfaction and their possible influences on intent to turnover. Revista de Gestão, 25(1), 84–101. 10.1108/REGE-12-2017-008

[R25] Mitchell TR, Holtom BC, Lee TW, Sablynski CJ, & Erez M (2001). Why people stay: Using job embeddedness to predict voluntary turnover. Academy of Management Journal, 44(6), 1102–1121. 10.5465/3069391

[R26] Moynihan DP, & Landuyt N (2008). Explaining turnover intention in state government: Examining the roles of gender, life cycle, and loyalty. Review of Public Personnel Administration, 28(2), 120–143. 10.1177/0734371X08315771

[R27] Moynihan DP, & Pandey SK (2007). The ties that bind: Social networks, person-organization value fit, and turnover intention. Journal of Public Administration Research and Theory, 18, 205–227. 10.1093/jopart/mum013

[R28] Park TY, & Shaw JD (2013). Turnover rates and organizational performance: A meta-analysis. Journal of Applied Psychology, 98(2), 268–309. 10.1037/a0030723 23244224

[R29] Partnership for Public Service. (2014). Publications and media library: Fed figures 2014–Federal departures. https://ourpublicservice.org/wp-content/uploads/2014/08/625ee3558139333eccbe73c47bcf941a-1414507030.pdf

[R30] Partnership for Public Service. (2019). Government-wide analysis: Overall findings and private sector comparison. https://bestplacestowork.org/analysis/

[R31] Pitts D, Marvel J, & Fernandez S (2011). So hard to say good-bye? Turnover intention among U.S. Federal Employees. Public Administration Review, 71(5), 751–760. 10.1111/j.1540-6210.2011.02414.x

[R32] Pourshaban D, Basurto-Dávila R, & Shih M (2015). Building and sustaining strong public health agencies. Journal of Public Health Management and Practice, 21, S80–S90. 10.1097/phh.0000000000000311 26422498

[R33] Rodwell JJ, Noblet AJ, & Allisey AF (2011). Improving employee outcomes in the public sector: The benefits of social support at work and job control. Personnel Review, 40(3), 383–397. 10.1108/00483481111118676

[R34] Saks AM (2006). Antecedents and consequences of employee engagement. Journal of Managerial Psychology, 21(7), 600–619. 10.1108/02683940610690169

[R35] Schaufeli WB, & Bakker AB (2004). Job demands, job resources, and their relationship with burnout and engagement: A multi-sample study. Journal of Organizational Behavior, 25(3), 293–315. 10.1002/job.248

[R36] Shaw JD (2011). Turnover rates and organizational performance: Review, critique, and research agenda. Organizational Psychology Review, 1(3), 187–213. 10.1177/2041386610382152

[R37] Shaw JD, Gupta N, & Delery JE (2005). Alternative conceptualizations of the relationship between voluntary turnover and organizational performance. Academy of Management Journal, 48, 50–68. 10.5465/AMJ.2005.15993112

[R38] Strober MH (1990). Human capital theory: Implications for HR managers. Industrial Relations, 29, 214–239. 10.1111/j.1468-232X.1990.tb00752.x

[R39] Trahant B (2009). Driving better performance through continuous employee engagement. Public Manager, 38(1), 54–58.

[R40] Trottier T, Van Wart M, & Wang X (2008). Examining the nature and significance of leadership in government organizations. Public Administration Review, 68(2), 319–333. 10.1111/j.1540-6210.2007.00865.x

[R41] U.S. Bureau of Labor Statistics. (2015). Economic news release: Table 2. Employment by major industry sector https://www.bls.gov/news.release/ecopro.t02.htm

[R42] U.S. Bureau of Labor Statistics. (2017). Economic news release: Job openings and labor turnover. https://www.bls.gov/news.release/jolts.htm

[R43] U.S. Bureau of Labor Statistics. (2019). Service-providing industries. https://www.bls.gov/iag/tgs/iag07.htm

[R44] U.S. Government Accountability Office. (2015). Federal workforce: Additional analysis and sharing of promising practices could improve employee engagement and performance (Report no. GAO-15–585).

[R45] U.S. Office of Personnel Management. (2014). Federal Employee Viewpoint Survey results: Appendix A.

[R46] U.S. Office of Personnel Management. (2015a). Engaging the federal workforce: How to do it and prove it. https://admin.govexec.com/media/gbc/docs/pdfs_edit/engaging_the_federal_workforce_white_paper.pdf

[R47] U.S. Office of Personnel Management. (2015b). Federal Employee Viewpoint Survey results: Government wide management report.

[R48] U.S. Office of Personnel Management. (2015c). Federal Employee Viewpoint Survey results: Report by agency.

[R49] U.S. Office of Personnel Management. (2015d). Federal Employee Viewpoint Survey results: Technical report.

[R50] U.S. Office of Personnel Management. (2015e). Federal employment documentation: Historical federal workforce tables. https://www.opm.gov/policy-data-oversight/data-analysis-documentation/federal-employment-reports/historical-tables/executive-branch-civilian-employment-since-1940/

[R51] U.S. Office of Personnel Management. (2016a). Building an engaging workplace: Understanding and using engagement drivers. https://www.opm.gov/fevs/archive/2016FILES/Engagement_Drivers_Background_and_Summary.pdf

[R52] U.S. Office of Personnel Management. (2016b). Federal Employee Viewpoint Survey: Government-wide management report.

[R53] U.S. Office of Personnel Management. (2018). FedScope: Federal human resources data. https://www.fedscope.opm.gov/

[R54] Vincent-Höper S, Muser C, & Janneck M (2012). Transformational leadership, work engagement, and occupational success. Career Development International, 17(7), 663–682. 10.1108/13620431211283805

[R55] Xu J, & Cooper Thomas H (2011). How can leaders achieve high employee engagement? Leadership & Organization Development Journal, 32(4), 399–416. 10.1108/01437731111134661

